# Antisense transcription in *Pseudomonas aeruginosa*

**DOI:** 10.1099/mic.0.000664

**Published:** 2018-05-08

**Authors:** Denitsa Eckweiler, Susanne Häussler

**Affiliations:** ^1^​ Institute of Molecular Bacteriology, TWINCORE, Centre of Experimental and Clinical Infection Research, Hannover, a joint venture of the Hannover Medical School and the Helmholtz Centre for Infection Research, Braunschweig, Germany; ^2^​ Department of Molecular Bacteriology, Helmholtz Centre for Infection Research, Braunschweig, Germany; ^†^​ Present address: Institute of Microbiology and Braunschweig Integrated Centre of Systems Biology, Braunschweig, Germany.

**Keywords:** antisense transcription, antisense RNA regulation by alternative sigma factors

## Abstract

A large number of antisense transcripts have been detected in diverse microbial genomes and considerable effort has been devoted to elucidating the functional role of antisense transcription. In this study, we reanalysed extensive RNA sequencing data from the opportunistic pathogen *Pseudomonas aeruginosa* and found that the majority of genes have a propensity for antisense transcription. Although antisense transcripts were found in more than 80 % of the genes of the *P. aeruginosa* genome, the majority of sequencing reads were mapping sense and only a minority (<2 %) were mapping antisense to genes. Similarly to the sense expression levels, the antisense expression levels varied under different environmental conditions, with the sense and antisense expression levels often being inversely regulated and modulated by the activity of alternative sigma factors. Environment-modulated antisense transcription showed a bias towards being antisense to genes within regions of genomic plasticity and to those encoding small regulatory RNAs. In the future, the validation and functional characterization of antisense transcripts, and novel transcripts that are antisense to small regulatory RNAs in particular, have the potential to contribute to our understanding of the various levels of transcriptional regulation and its dynamics in the bacterial pathogen *P. aeruginosa*.

## Introduction

With the emergence of high-throughput RNA sequencing approaches, an unexpected number (several hundred) of antisense (AS) RNA transcripts have been detected in diverse microbial genomes [1–5]. Increasing information on the abundance, size and genomic localization of asRNA is currently being acquired [5–11]. However, it is still a matter of debate as to whether highly abundant asRNAs are functionally relevant [12–15].

RNAs are well recognized as having important regulatory roles in bacterial physiology and pathogenicity [11, 16]. Next to *trans*-encoded small regulatory RNAs, whose target is located elsewhere on the chromosome [17], RNA elements that are present in the 5′ UTR of target mRNAs (e.g. thermosensors and riboswitches) have regulatory roles [18–21]. Furthermore, the expression of asRNA offers huge potential for regulatory transcription through the mechanisms of transcription interference and transcription attenuation [22–24]. There are increasing reports of *cis*-encoded regulatory asRNAs that overlap and are thus perfectly complementary to their target genes encoded on the opposite strand of the same genomic locus [25].

In this study we aimed to explore antisense transcription in the opportunistic pathogen *Pseudomonas aeruginosa.* We reanalysed previously recorded RNA-sequencing datasets [26, 27] and found transcription of both DNA strands in large parts of the genome. However, overall, fewer than 2 % of the mRNA sequencing reads mapped antisense to genes. The large majority of asRNA did not exhibit differential transcript abundance, even if the bacteria were cultured under changing environmental conditions. Nevertheless, a sub-fraction of the antisense transcripts (<5 %), many of which were found to be antisense to small regulatory RNAs, varied under different environmental conditions or upon the modulation of alternative sigma factor gene expression. For most of those asRNA transcripts, an inverse relation of their expression to that of the corresponding sense mRNA was observed.

## Methods

### Data availability

The RNA sequencing data for the *P. aeruginosa* PA14 reference strain cultivated under 14 different growth conditions (a total of 51 samples, environmental dataset) were generated in the context of a previous study [26]. They are accessible as a single dataset from the Gene Expression Omnibus (GEO) database under accession number GSE55197. The sigma factor transcriptomic and ChIP-seq data have also been published previously [27] and are accessible from the GEO database (accession numbers GSE54997 and GSE54998 united under SuperSeries GSE54999). The RNA was prepared according to the published protocols, which involve purification of the samples by the use of MinElute columns (Qiagen), which have a cutoff of 70 bp.

### Data normalization and sensitivity limit

For all calculations, the number of reads falling within the gene coordinates was used, where the left, 5′ end of the read must be placed within the gene boundaries in order to be included in the gene pileup. The absolute gene read counts (separate sense and antisense pileups) in the environmental dataset were normalized and *log_2_*-transformed to yield normalized reads per kilobase of gene sequence (nRPK) values as previously described [26].

$$nRPK_{ij}=log_{2}\left(\frac{1000}{l_{i}}\times \frac{RPG_{i}}{F_{j}}+1\right)$$

nRPK*_ij_* is the number of normalized reads per kilobase of gene sequence of gene *i* and expression profile *j*, *l_i_* is the length of gene *i*, RPG*_i_* is the number of reads that mapped to the locus of gene *i* and *F_j_* is the size factor (calculated by DESeq) of expression profile *j*. Calculations were performed separately for the sense and antisense datasets. We defined a sensitivity limit for the antisense gene expression, normalized Reads Per Kilobase of transcript, nRPK_0_, to be equal to 3.15, based on the sample showing the lowest sequencing depth (i.e. the expression profile with the lowest size factor as calculated by DESeq [28]). The nRPK_0_ of the sense expression dataset was 3.62.

### Calculation of differential sense and antisense transcription

The R package DESeq [28] was used for differential sense and antisense gene expression analysis. Both sense and antisense expression comparisons were performed using the gene read pileups and considering reads falling within the gene coordinates. For each comparison, the biological replicates for each condition were used. For the 14 growth conditions the number of comparisons amounted to n*(*n*−1)/2, or altogether 91 comparisons. The Benjamini and Hochberg correction was used to control the false-discovery rate (FDR) at 5 % to determine the list of differentially regulated transcripts. Sense and antisense transcription of genes were identified as differentially expressed if they were at least twofold regulated between the two conditions and their Benjamini–Hochberg corrected *P* value was maximally 0.05.

### Calculation of enrichment factor

Using the *Pseudomonas* Community Annotation Project (PseudoCAP) annotation available for *P. aeruginosa* PA14, over- or underrepresentation was calculated by comparing normalized PseudoCAP classes experimentally detected and normalized PseudoCAP classes annotated using the following equation: EF=(number of genes of a specific PseudoCAP class detected/number of all detected genes in the analysis)/(genome-wide number of all genes belonging to that specific PseudoCAP class/number of all genes with PseudoCAP class annotation). Detailed information on the enrichment factor (EF) calculation is available in [29].

### Assigning autonomous antisense RNA to primary sigma factor regulons

In order to identify alternative sigma factors that regulate the expression of antisense transcripts, we performed a motif search in the region of the transcriptional start site (TSS) of those antisense transcripts that were not part of the 3′ untranslated region (UTR) or the 5′UTR of the sense transcripts (autonomous transcripts). The promoter was defined as the region spanning 200 bp upstream and 100 bp downstream of the TSS of the asRNA, as detected by a custom Perl script. Briefly, this script takes as input the list of genes defined to be differentially regulated, as well as the genomic read pileup for the sense and antisense strands. Once the gene coordinates have been allocated, the script records the antisense read distribution throughout the gene; using the gene orientation information, the script searches for the transcript end that is determined as soon as the read coverage drops under a given cutoff – here it was set to five reads. Going back from this position, the script assigns the transcript start using the supplied read cutoff information, as well as the read pileup increase/drop in a 50-bp window. We made use of the fact that *P. aeruginosa* TSSs are very sharp peaks and the read pileup usually drops critically after one read length. The TSS is initially detected once the read pileup at a position is at least three times higher than the average pileup in the window. Finally, the transcript TSS is iteratively allocated at 5 bp resolution in the 50-bp window. Having determined the transcript TSS, we used the position weight matrices published in [27] for the motif scan with the MAST module of the MEME suite [30]. We also searched for ChIP-seq-enriched peaks in those promoter regions using the data from our previous study [27]. Transcripts were defined to be a member of an alternative sigma factor regulon if they exhibited sigma factor-dependent transcript regulation and fulfilled at least one of the following criteria: (i) their promoter was enriched in ChIP-seq experiments and (ii) their promoter contained a sigma factor-binding motif.

## Results

### Global survey of *P. aeruginosa* antisense transcripts

We previously recorded strand-specific single-nucleotide-resolution mRNA sequence data for the *P. aeruginosa* type strain PA14 grown under 14 different environmental conditions. Overall, 51 transcriptional profiles of PA14 were obtained [26]. The distribution of normalized gene expression for all transcribed PA14 genes indicated that almost the entire genome of *P. aeruginosa* PA14 is transcriptionally active above the calculated sensitivity limit under at least one environmental condition [26]. In this study we focused on the expression of antisense RNA. Sequencing reads were found to be antisense to 4993 PA14 genes (>80 % of the PA14 genes) above the sensitivity limit under at least 1 environmental condition. Although our data indicate that for the great majority of genes an asRNA transcript can be detected, only 1.69 % of all the sequencing reads that mapped to the PA14 genome mapped antisense to the PA14 genes. This is consistent with the results from a previous study [31], where on average 2.6 % of the reads mapped antisense to known transcripts. Of note, among the 82 PA14 genes that have been previously reported to be never transcribed under any of the given environmental conditions [26], 63 showed antisense transcription.

### A minority of antisense transcripts is differentially regulated under changing environmental conditions

**Fig. 1.**
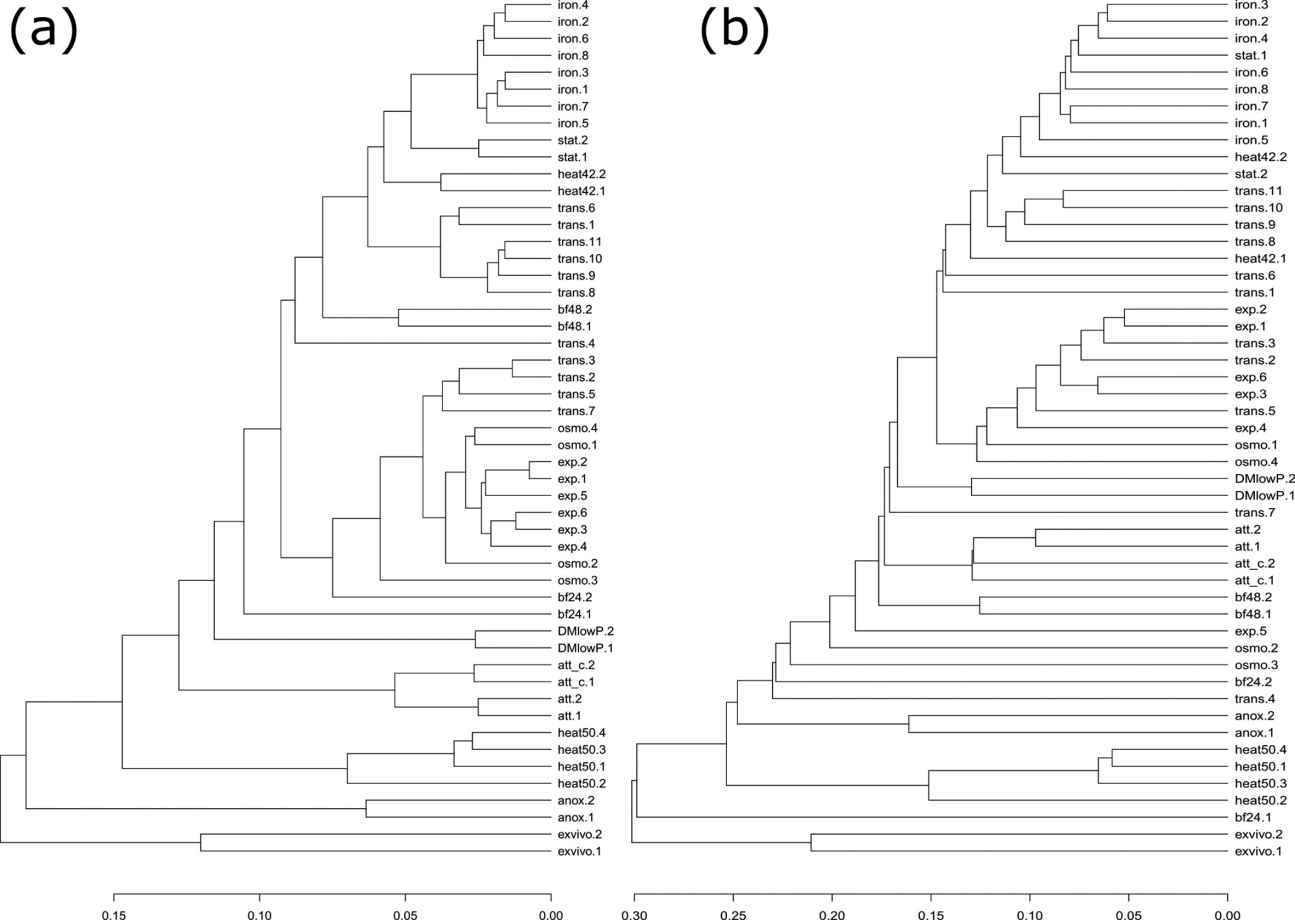
Dendrograms showing hierarchical clustering of the sense (a) and antisense (b) samples. The growth conditions are as follows: anoxic cultivation (anox), attached cells (att), nonattached population in attachment expreriment (att_c), anoxic cultivation (anox), 24-h-old static biofilm (bf24), 48-h-old biofilm (bf48), heat shock at 42° (heat42) or 50° (heat50), mouse tumour infection model (*ex vivo*), exponential (exp), late exponential (trans), stationary phase (stat), iron deficiency (iron) and low osmolarity (osmo).

Pairwise comparisons of the transcriptional profiles of PA14 cultivated under 14 different environmental conditions revealed a differential in sense gene expression in 3111 genes under at least 1 of the culture conditions [26] (Fig. S1, available in the online version of this article). Fig. 1(a) shows that transcriptional profiles recorded under the same culture conditions cluster well, confirming the high reproducibility of the replicates.

**Fig. 2.**
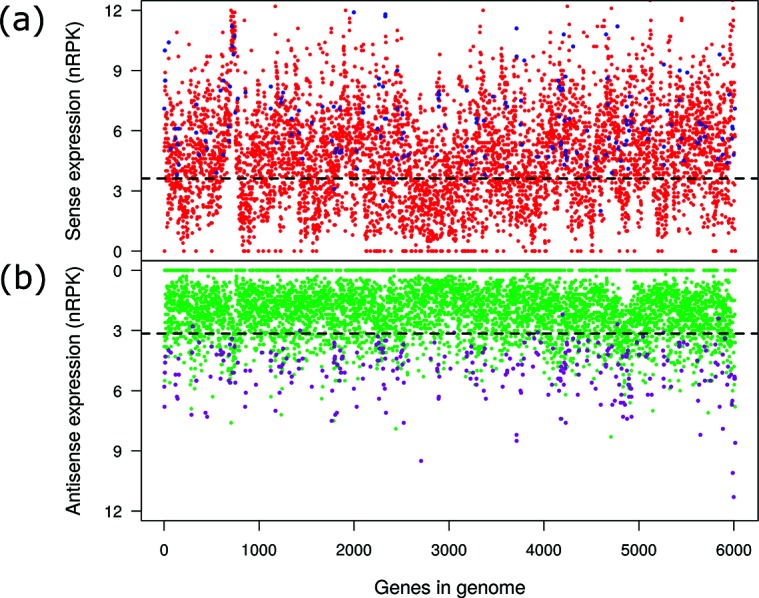
Sense (a) and antisense (b) gene expression of the *P. aeruginosa* genome under 14 different growth conditions (based on overall 51 transcriptional profiles). Each dot shows the median sense (red, (a)) and antisense (green, (b)) expression values (*log_2_*-transformed normalized expression values, nRPK) for each of the >6000 genes. The black dashed lines show the sensitivity limit of the expression detection (nRPK_0_=3.15 for the antisense (b) and nRPK_0_=3.62 for the sense (a) data; for details please see the Methods section). Two hundred and ninety-eight genes have an antisense transcript that is differentially regulated under changing conditions (coloured in magenta), and 186 of them also exhibit a differential in sense gene expression (blue dots). The genes (*x*-axis) are ordered as they are found on the chromosome.

Interestingly, the expression profiles of the asRNAs also varied with external stimuli. However, of the 4993 genes with antisense transcripts, only 298 (6.0 %; Table S1, Fig. S1) had an antisense transcript that was differentially regulated under the various environmental conditions (Fig. 2). One hundred and eighty-six (62 %; Table S1) of these genes also exhibited a differential in the expression of the corresponding sense RNAs (Fig. 2). Of note, the antisense transcriptional profiles that were recorded under the same culture conditions also clustered (Fig. 1b). This indicates that the environment-driven differential in antisense transcription is also reproducible, albeit to lower levels than for the sense transcriptional profiles.

Fifty-eight of these antisense transcripts (19.5 %) were oriented antisense to genes lying in regions of genomic plasticity (RGPs), while only 8.4 % of all the genes in PA14 were found in RGPs, resulting in an EF of 2.32 (58/298 vs 503/6015 genes, Table 1). In accordance with these results, a previous study [32] found fivefold more asRNAs in the pathogenicity island PAPI-1 as compared to antisense RNAs found in the core genome. We also assigned the 298 differentially expressed antisense transcripts to functional classes (Table 1). Among the functional categories that exhibited a greater than 1.5-fold enrichment we found genes related to phage, transposon or plasmid (EF: 1.94), and genes involved in processes such as adaptation and protection (EF: 1.58). Remarkably, there was a particularly strong enrichment of transcripts that were antisense to small regulatory RNAs (EF: 6.13). This was true for the 298 genes exhibiting a differential expression of the antisense transcripts, as well as for the 186 genes with a differential in both sense and antisense transcripts.

**Table 1.** Enrichment of gene targets of antisense RNA within PseudoCAP functional classes The table lists the enrichment factors calculated by comparing the numbers of genes belonging to a functional class that exhibit an anti-sense transcript (in parentheses) against the total number of genes belonging to this functional class. Asterisks denote significantly overrepresented (*P*<0.05) functional classes.

PseudoCAP functional class	Enrichment factor and number (in parentheses) of antisense transcripts exhibiting environment-driven differential expression
Motility/attachment	1.27 (9)
Transcriptional regulators	1.33* (32)
Hypothetical	1.46* (100)
Adaptation/protection	1.58* (16)
Related to phage, transposon or plasmid	1.94* (9)
Genes from regions of genomic plasticity (RGPs)	2.32* (503)
Non-coding RNA gene (sRNA)	6.13* (12)

### Alternative Sigma factors modulate the expression of antisense transcripts in *P. aeruginosa*

**Fig. 3.**
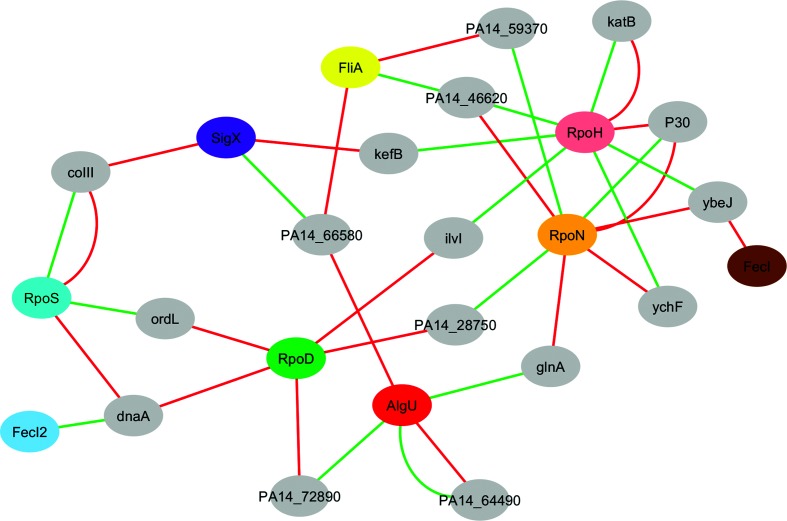
Reconstruction of the combination of sigma factors that govern expression of the sense and antisense transcripts of the indicated genes (Table S2). The lines connect each gene (grey ellipse) to the sigma factor that regulates sense (red line) and antisense (green line) transcription of the gene.

We previously published data on the impact of 10 alternative sigma factors (RpoN, RpoH, RpoS, SigX, FliA, RpoH, AlgU, PvdS, FpvI and FecI2) on the global transcriptional profile in the type strain PA14 [27]. We have also inactivated these alternative sigma factors (with the exception of RpoH and FecI2) and recorded transcriptional profiles under growth conditions that are expected to support sigma factor-dependent gene expression [27]. Here, we re-evaluated the impact of inactivation/overexpression of the alternative sigma factors and concentrated our analysis on the antisense transcriptional profile of those 298 genes whose antisense transcription was changed under different environmental conditions (Fig. 1). We found 45 genes with antisense transcripts that also exhibited differential expression upon the modulation of alternative sigma factor expression (Fig. S1 and Table S2). Thirty of these antisense transcripts could be assigned to a particular antisense primary sigma factor regulon [27]. In 17 of them both sense/antisense transcripts could be assigned. Fig. 3 depicts which gene transcripts (sense and antisense) are governed by which sigma factors. In four genes the sense and antisense transcripts were governed by the same sigma factor.

### Comparison to previous studies

Three studies [31–33] have previously investigated antisense transcription in *P. aeruginosa*. One of these studies [32] identified 384 antisense transcriptional start sites in PA14 grown at 28 and 37 °C with overlapping transcription on the reverse DNA strand. In another study [33], the strains PAO1 and PA14 were subjected to antisense transcriptional profiling following growth in brain heart infusion (BHI) medium until the early stationary phase. Overall, 60 asRNAs were identified. However, not all of them were found in both strain backgrounds, indicating that there is a strain-specific expression of antisense transcripts. In the third study [31], 232 novel asRNAs were detected, of which 18 had been detected in 1 and 4 had been detected in both of the previous antisense detection studies. With our study, the number of *P. aeruginosa* antisense transcripts that have been identified in at least 2 independent studies increased from 33 to 134 (Table S3 and Fig. S2).

## Discussion

Much remains to be learned about whether and to what extent antisense transcription serves the maintenance of the structural integrity of the chromosome, facilitates the emergence of new RNA genes or drives gene regulatory functions. In this study, we re-evaluated previously recorded extensive RNA-seq data and focused on the expression of antisense transcripts in the opportunistic pathogen *P. aeruginosa*. We found that antisense transcription in *P. aeruginosa* is widespread, as has been observed in other bacterial genomes previously [5, 34, 35]. Sequencing reads that were antisense to more than 80 % of the genes within the PA14 genome could be detected.

Although antisense transcripts were found throughout the *P. aeruginosa* genome, their expression levels were generally very low. This could argue for a more general function, as a spurious global transcription might secure genomic regions from being completely silenced and thus might aid the stabilization and maintenance of the structural integrity of the chromosome. Interestingly, for many (63) of the genes that had never been transcribed under any of the 14 previously tested environmental conditions (82 genes had never been transcribed [26]), antisense sequencing reads could be detected.

Furthermore, although the expression levels of the antisense RNA were generally very low, the expression levels of some antisense transcripts (6 %) changed under different environmental conditions, as had been observed previously, e.g. in *Bacillus subtilis* and *Staphylococcus aureus* cultures [36, 37]. The sense and antisense expression levels were often inversely regulated, implying a gene regulatory function, as antisense transcripts can regulate the conjugated sense transcript negatively and interfere with gene expression. Intriguingly, many of the differentially regulated antisense transcripts were antisense to genes within regions of genomic plasticity and genes encoding for regulatory RNAs. This indicates that regulatory RNAs may not only exert their activity via RNA interference, but they could also themselves be subject to regulation through RNA–RNA interaction.

We also demonstrate that antisense transcripts are modulated by the activity of alternative sigma factors. Bacterial sigma factors are subunits of the RNA polymerase that mediate bacterial adaptation to changing and stressful habitats. The level and activity of alternative sigma factors are highly regulated and vary depending on the environmental conditions [38, 39]. Regulation of antisense transcripts by alternative sigma factors therefore implies that asRNAs might have regulatory roles and contribute to bacterial adaptation [36].

The fact that asRNA detection is strongly influenced by not only the strain background [33], the applied RNA-seq technology and the bioinformatics analysis, but also the culture conditions, restricts studies on the physiological role of asRNA transcripts. Nevertheless, the validation and functional characterization of novel asRNAs remains an important task. In this context, a more detailed analysis of RNAs that are antisense to small regulatory RNAs might make an particularly strong contribution to our understanding of the transcriptional boundaries and dynamics of bacterial pathogens [40].

## Supplementary Data

1024 Supplementary File 1
