# Supplementary material for: Antisense transcription in Pseudomonas aeruginosa
Source: Microbiology (Reading). 2018 May 8;164(6):889–95. doi: 10.1099/mic.0.000664 (PMC6097033; doi:10.1099/mic.0.000664)
Supplement: Supplementary File 1 [file mic-164-889-s001.pdf]

Supplementary Material

Antisense transcription in *Pseudomonas aeruginosa*

Denitsa Eckweiler and Susanne Häussler

Fig. S1

Fig. S2

Table S1

Table S2

Table S3

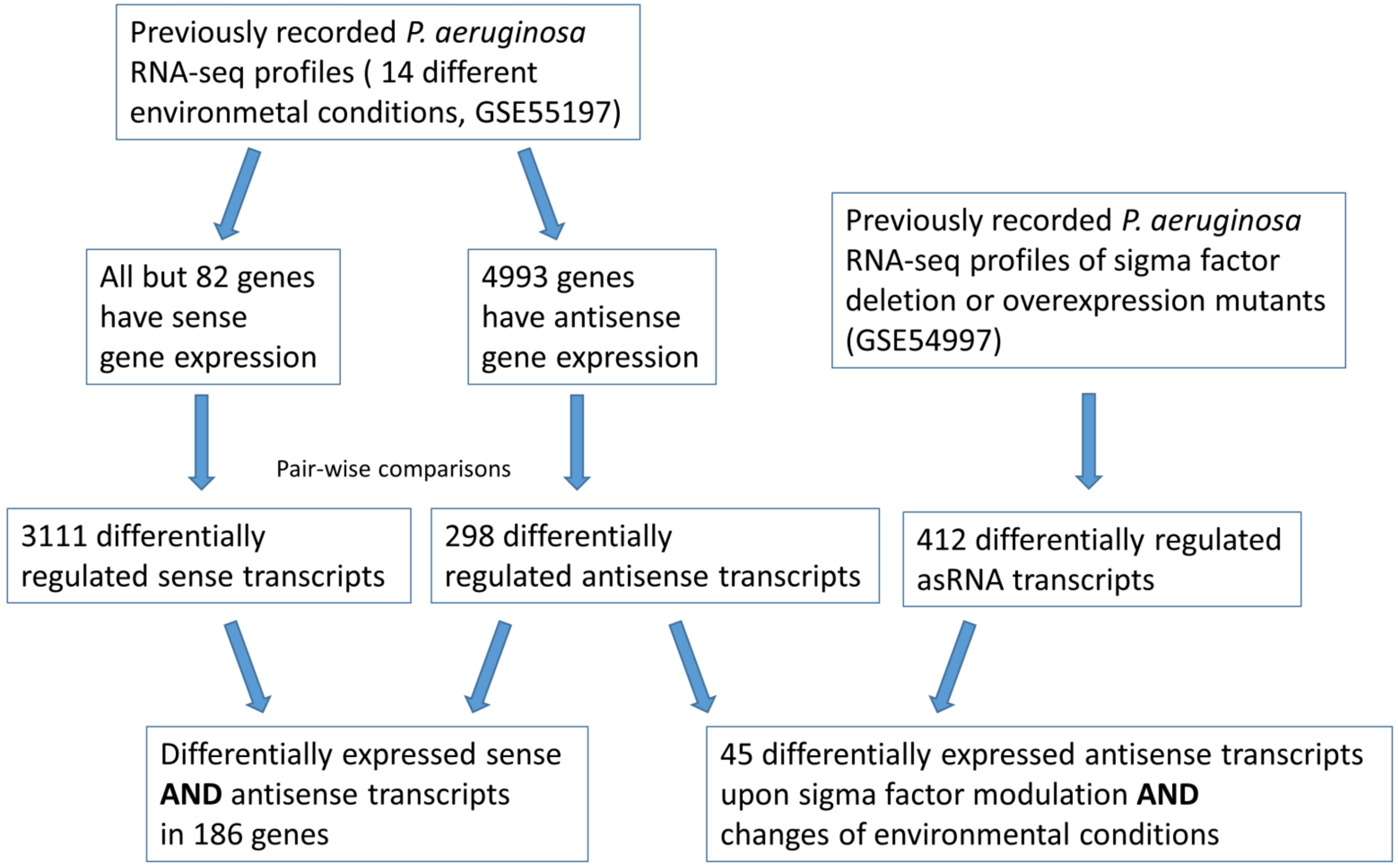

Fig. S1. Analysis workflow in the present work. All recorded data are publicly available.

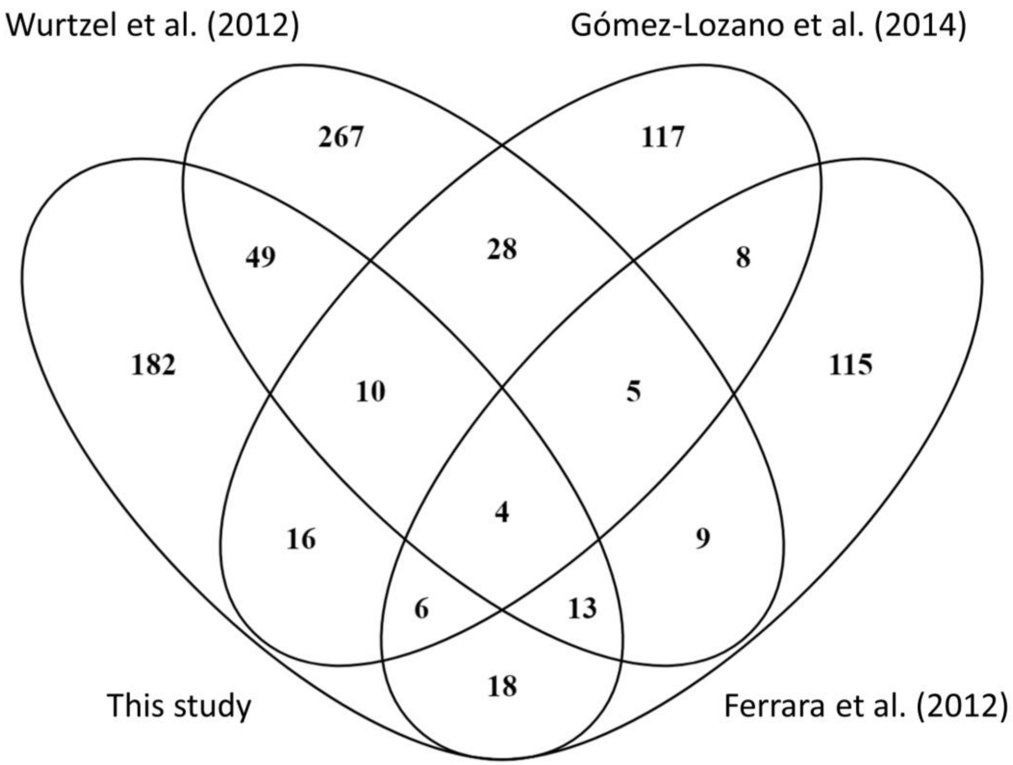

Fig. S2. Comparison between the genes having antisense RNAs found in this study and three previous works.

Table S1. Genes having antisense transcripts that were differentially regulated under changing environmental conditions

Gene locus	Sense regulation	Gene name	PseudoCap class
PA0296-PA0297	y	*P1*	Non-coding RNA gene (sRNA)
PA0836.1		*P5*	Non-coding RNA gene (sRNA)
PA1052-PA1053	y	*sRNA622*	Non-coding RNA gene (sRNA)
PA14_00010	y	*dnaA*	DNA replication, recombination, modification and repair
PA14_00050	y	*gyrB*	DNA replication, recombination, modification and repair
PA14_00480	y		Hypothetical, unclassified or unknown
PA14_00730	y		Hypothetical, unclassified or unknown
PA14_01320	y	*coIII*	Energy metabolism
PA14_01330	y		Membrane proteins
PA14_01500	y		Transcriptional regulators
PA14_01600	y	*mmsA*	Energy metabolism; Putative enzymes; Carbon compound catabolism
PA14_01720	y	*ahpF*	Adaptation / Protection
PA14_01770	y		Membrane proteins
PA14_03420			Hypothetical, unclassified or unknown
PA14_03530			Transcriptional regulators
PA14_03610		*ycaL*	Chaperones / Heat shock proteins
PA14_04080	y	*yecS*	Membrane proteins; Transport of small molecules
PA14_04110	y	*serA*	Amino acid biosynthesis and metabolism
PA14_04490	y		Hypothetical, unclassified or unknown
PA14_05310	y	*gshB*	Amino acid biosynthesis and metabolism; Biosynthesis of cofactors, prosthetic groups and carriers
PA14_05520	y	*mexR*	Transcriptional regulators
PA14_06030	y		Fatty acid and phospholipid metabolism
PA14_06160		*fiuA*	Transport of small molecules; Membrane proteins
PA14_06870	y	*dnr*	Transcriptional regulators
PA14_07370	y		Membrane proteins

PA14_07380			Hypothetical, unclassified or unknown
PA14_07440			Hypothetical, unclassified or unknown
PA14_07730	y	*ksgA*	Transcription, RNA processing and degradation
PA14_07770	y	*ostA*	Adaptation / Protection
PA14_08390	y	*speD*	Central intermediary metabolism
PA14_08440	y		Carbon compound catabolism; Putative enzymes
PA14_08520		*anmK*	Translation, post-translational modification and degradation
PA14_08540	y		Cell wall / LPS / capsule
PA14_08750	y	*rplL*	Translation, post-translational modification and degradation
PA14_08850	y	*rplC*	Translation, post-translational modification and degradation
PA14_08940	y	*rpsQ*	Translation, post-translational modification and degradation
PA14_09050	y	*secY*	Membrane proteins; Protein secretion / Export apparatus
PA14_09160	y	*bfrA*	Transport of small molecules; Adaptation / Protection
PA14_09490	y	*phzM*	Putative enzymes
PA14_10480			Transcriptional regulators
PA14_10540	y	*fixG*	Energy metabolism; Putative enzymes
PA14_10570		*hpcH*	Carbon compound catabolism; Putative enzymes
PA14_11120		*rcsB*	Transport of small molecules; Transcriptional regulators
PA14_11880			Hypothetical, unclassified or unknown
PA14_12700			Membrane proteins
PA14_13920			Hypothetical, unclassified or unknown
PA14_13940	y		Adaptation / Protection; Secreted factors (toxins, enzymes, alginate)
PA14_13950	y		Hypothetical, unclassified or unknown
PA14_14000			Transcriptional regulators; Amino acid biosynthesis and metabolism
PA14_14560			Hypothetical, unclassified or unknown
PA14_14680	y	*suhB*	Translation, post-translational modification and degradation; Adaptation / Protection
PA14_15090			Membrane proteins
PA14_15140			Hypothetical, unclassified or unknown
PA14_15350	y		Related to phage, transposon or plasmid
PA14_15360	y		Hypothetical, unclassified or unknown

PA14_15520		*trbJ*	Related to phage, transposon or plasmid
PA14_15580	y		Putative enzymes
PA14_16010			Fatty acid and phospholipid metabolism
PA14_16380			Transcriptional regulators
PA14_16670	y	*cadR*	Transcriptional regulators
PA14_16680	y		Hypothetical, unclassified or unknown
PA14_17590	y		Hypothetical, unclassified or unknown
PA14_17600	y		Protein secretion / Export apparatus
PA14_17720			Transcriptional regulators
PA14_18080	y		Transcriptional regulators; Antibiotic resistance and susceptibility
PA14_19370	y	*asnB*	Amino acid biosynthesis and metabolism
PA14_20700	y		Membrane proteins
PA14_21210			Membrane proteins
PA14_21840	y		Membrane proteins
PA14_21900			Putative enzymes
PA14_22080	y		Hypothetical, unclassified or unknown
PA14_22130			Hypothetical, unclassified or unknown
PA14_22140	y		Hypothetical, unclassified or unknown
PA14_22180			Hypothetical, unclassified or unknown
PA14_22240			Hypothetical, unclassified or unknown
PA14_22270	y		Hypothetical, unclassified or unknown
PA14_22480	y		Hypothetical, unclassified or unknown
PA14_22540		*wrbA*	Energy metabolism
PA14_22800	y	*yciB*	Cell division; Membrane proteins
PA14_22870	y		Hypothetical, unclassified or unknown
PA14_22880	y		Hypothetical, unclassified or unknown
PA14_23060	y	*hexR*	Carbon compound catabolism; Transcriptional regulators
PA14_23100		*mutT*	DNA replication, recombination, modification and repair
PA14_23200		*yciK*	Putative enzymes
PA14_23470		*wbpM*	Putative enzymes; Membrane proteins; Cell wall / LPS / capsule

PA14_24650	y	*rmf*	Translation, post-translational modification and degradation
PA14_24990	y		Hypothetical, unclassified or unknown
PA14_25030	y		Hypothetical, unclassified or unknown
PA14_25800	y		Transcriptional regulators
PA14_25840	y		Energy metabolism
PA14_26870			Hypothetical, unclassified or unknown
PA14_27630		*rebB1*	Hypothetical, unclassified or unknown
PA14_27770	y	*yadG*	Transport of small molecules
PA14_27830			Transcriptional regulators; Adaptation / Protection
PA14_27920		*vacJ*	Fatty acid and phospholipid metabolism; Antibiotic resistance and susceptibility
PA14_27940	y		Transcriptional regulators; Two-component regulatory systems
PA14_27950	y		Two-component regulatory systems
PA14_28020			Hypothetical, unclassified or unknown
PA14_28180	y	*ordL*	Energy metabolism; Carbon compound catabolism
PA14_28390			Membrane proteins
PA14_28400	y	*oprQ*	Membrane proteins; Transport of small molecules; Motility / Attachment; Antibiotic resistance and susceptibility
PA14_28410	y		Hypothetical, unclassified or unknown
PA14_28470			Hypothetical, unclassified or unknown
PA14_28610	y		Hypothetical, unclassified or unknown
PA14_28670	y	*rpmI*	Translation, post-translational modification and degradation
PA14_28680	y	*rplT*	Translation, post-translational modification and degradation
PA14_28750			Hypothetical, unclassified or unknown
PA14_28770	y		Hypothetical, unclassified or unknown
PA14_29600	y	*ptpS*	Biosynthesis of cofactors, prosthetic groups and carriers
PA14_30110	y	*purB*	Amino acid biosynthesis and metabolism; Nucleotide biosynthesis and metabolism
PA14_30200	y	*cspD*	Transcriptional regulators; Adaptation / Protection
PA14_30240	y	*infA*	Translation, post-translational modification and degradation
PA14_30600			Transport of small molecules; Membrane proteins
PA14_30700			Transcriptional regulators; Two-component regulatory systems

PA14_30800	y		Energy metabolism
PA14_30830	y		Transcriptional regulators; Two-component regulatory systems
PA14_30960		*traG*	Related to phage, transposon or plasmid
PA14_30980	y		Hypothetical, unclassified or unknown
PA14_30990	y		Hypothetical, unclassified or unknown
PA14_31560	y		Transcriptional regulators
PA14_33360			Hypothetical, unclassified or unknown
PA14_34670			Putative enzymes
PA14_35150			Energy metabolism; Putative enzymes
PA14_35540	y	*bkdR*	Transcriptional regulators
PA14_35550		*pslO*	Hypothetical, unclassified or unknown
PA14_35710	y		Hypothetical, unclassified or unknown
PA14_35720	y		Hypothetical, unclassified or unknown
PA14_35750	y	*tpnC*	Related to phage, transposon or plasmid
PA14_35800	y		Hypothetical, unclassified or unknown
PA14_36470	y		Hypothetical, unclassified or unknown
PA14_37200			Hypothetical, unclassified or unknown
PA14_37730			Transport of small molecules; Membrane proteins
PA14_39420	y		Hypothetical, unclassified or unknown
PA14_39460	y		Hypothetical, unclassified or unknown
PA14_39480	y		Hypothetical, unclassified or unknown
PA14_39980		*qscR*	Transcriptional regulators
PA14_40070		*gst*	Central intermediary metabolism; Putative enzymes
PA14_40080	y		Hypothetical, unclassified or unknown
PA14_40220			Putative enzymes
PA14_40280	y		Hypothetical, unclassified or unknown
PA14_40740	y		Hypothetical, unclassified or unknown
PA14_40750	y		Hypothetical, unclassified or unknown
PA14_41563		*cobA*	Biosynthesis of cofactors, prosthetic groups and carriers
PA14_41680	y	*ydiA*	Hypothetical, unclassified or unknown

PA14_41820	y	*pabB*	Biosynthesis of cofactors, prosthetic groups and carriers
PA14_41830	y	*thrH*	Amino acid biosynthesis and metabolism
PA14_41960			Hypothetical, unclassified or unknown
PA14_42850	y	*folE*	Biosynthesis of cofactors, prosthetic groups and carriers
PA14_44110			Membrane proteins
PA14_44470	y	*hemN*	Biosynthesis of cofactors, prosthetic groups and carriers
PA14_44530		*ygdE*	Membrane proteins
PA14_44640	y		Hypothetical, unclassified or unknown
PA14_44650	y		Hypothetical, unclassified or unknown
PA14_45010		*hyi*	Central intermediary metabolism
PA14_45470			Central intermediary metabolism
PA14_45950	y	*rsaL*	Adaptation / Protection; Transcriptional regulators
PA14_45960	y	*lasR*	Adaptation / Protection; Transcriptional regulators
PA14_46150			Putative enzymes
PA14_46460	y		Hypothetical, unclassified or unknown
PA14_46530	y		Hypothetical, unclassified or unknown
PA14_46620	y		Putative enzymes
PA14_46720			Hypothetical, unclassified or unknown
PA14_46850			Transcriptional regulators
PA14_46910	y	*ybeJ*	Transport of small molecules
PA14_47500		*sseA*	Transport of small molecules; Putative enzymes
PA14_48030			Adaptation / Protection; Chemotaxis
PA14_48240			Transport of small molecules; Antibiotic resistance and susceptibility
PA14_48530			Putative enzymes
PA14_48700	y	*kefB*	Transport of small molecules
PA14_48770			Transcriptional regulators
PA14_49060			Transcription, RNA processing and degradation
PA14_50000	y		Hypothetical, unclassified or unknown
PA14_50510	y		Hypothetical, unclassified or unknown
PA14_50620	y		Hypothetical, unclassified or unknown

PA14_51240	y	*purC*	Nucleotide biosynthesis and metabolism
PA14_51340	y	*mvfR*	Biosynthesis of cofactors, prosthetic groups and carriers; Transcriptional regulators
PA14_51350	y	*phnB*	Amino acid biosynthesis and metabolism; Adaptation / Protection
PA14_51500			Hypothetical, unclassified or unknown
PA14_51530	y	*exoU*	Secreted factors (toxins, enzymes, alginate)
PA14_51540			Related to phage, transposon or plasmid
PA14_51550	y		Related to phage, transposon or plasmid
PA14_51590	y		Hypothetical, unclassified or unknown
PA14_51730	y	*tolA*	Membrane proteins; Transport of small molecules
PA14_51880	y	*oprD*	Transport of small molecules
PA14_51890			Hypothetical, unclassified or unknown
PA14_51940	y		Hypothetical, unclassified or unknown
PA14_51950	y		Hypothetical, unclassified or unknown
PA14_52060	y		Protein secretion / Export apparatus
PA14_52080	y		Hypothetical, unclassified or unknown
PA14_52090			Hypothetical, unclassified or unknown
PA14_52120	y		Hypothetical, unclassified or unknown
PA14_52260	y	*lemA*	Two-component regulatory systems
PA14_52290			Hypothetical, unclassified or unknown
PA14_52465			Hypothetical, unclassified or unknown
PA14_52580	y	*lysC*	Amino acid biosynthesis and metabolism
PA14_53070	y	*hpd*	Amino acid biosynthesis and metabolism
PA14_53570	y		Related to phage, transposon or plasmid
PA14_53650			Hypothetical, unclassified or unknown
PA14_53770			Hypothetical, unclassified or unknown
PA14_53780			Membrane proteins; Transport of small molecules
PA14_53800		*mntH2*	Membrane proteins; Transport of small molecules
PA14_54830	y		Putative enzymes
PA14_54850	y		Hypothetical, unclassified or unknown
PA14_54930	y		Putative enzymes

PA14_55410			Transport of small molecules
PA14_55820	y		Membrane proteins; Motility / Attachment
PA14_56170			Hypothetical, unclassified or unknown
PA14_56250			Putative enzymes
PA14_56560	y		Secreted factors (toxins, enzymes, alginate); Fatty acid and phospholipid metabolism
PA14_56570			Fatty acid and phospholipid metabolism
PA14_57250	y		Hypothetical, unclassified or unknown
PA14_57260	y	*lpxC*	Cell wall / LPS / capsule
PA14_57450	y	*mraW*	Cell wall / LPS / capsule
PA14_57500	y	*gmhA*	Carbon compound catabolism; Putative enzymes
PA14_57600	y		Energy metabolism; Putative enzymes
PA14_58000	y	*sodM*	Adaptation / Protection
PA14_58060			Hypothetical, unclassified or unknown
PA14_58380			Transcriptional regulators
PA14_58550	y	*lpxO1*	Putative enzymes; Cell wall / LPS / capsule
PA14_58730	y	*pilA*	Motility / Attachment
PA14_58800	y		Hypothetical, unclassified or unknown
PA14_58910			Related to phage, transposon or plasmid
PA14_59090	y		Hypothetical, unclassified or unknown
PA14_59160			Hypothetical, unclassified or unknown
PA14_59180			DNA replication, recombination, modification and repair
PA14_59350		*pilV2*	Motility / Attachment
PA14_59370			Hypothetical, unclassified or unknown
PA14_59410			Hypothetical, unclassified or unknown
PA14_59600	y		Hypothetical, unclassified or unknown
PA14_59760		*cupD5*	Motility / Attachment
PA14_59770	y	*rcsB*	Two-component regulatory systems
PA14_59780	y	*rcsC*	Two-component regulatory systems
PA14_59790	y	*pvrR*	Two-component regulatory systems
PA14_59830			DNA replication, recombination, modification and repair

PA14_59840	y		Hypothetical, unclassified or unknown
PA14_59850			Hypothetical, unclassified or unknown
PA14_59900			Hypothetical, unclassified or unknown
PA14_59960	y		Translation, post-translational modification and degradation
PA14_60020			Hypothetical, unclassified or unknown
PA14_60050	y		Related to phage, transposon or plasmid
PA14_60090	y		Hypothetical, unclassified or unknown
PA14_60290	y	*pilW*	Motility / Attachment
PA14_60310	y	*pilY1*	Motility / Attachment
PA14_60320	y	*pilE*	Motility / Attachment
PA14_61040		*katB*	Adaptation / Protection
PA14_61330		*mgtC*	Transport of small molecules
PA14_61820	y	*ychF*	Translation, post-translational modification and degradation
PA14_62160	y	*ilvI*	Biosynthesis of cofactors, prosthetic groups and carriers; Amino acid biosynthesis and metabolism
PA14_62370			Hypothetical, unclassified or unknown
PA14_62400	y	*yfdZ*	Transport of small molecules; Putative enzymes
PA14_62670	y		Hypothetical, unclassified or unknown
PA14_62880	y	*yhbY*	Translation, post-translational modification and degradation
PA14_64400			Hypothetical, unclassified or unknown
PA14_64440		*bcpA*	Carbon compound catabolism
PA14_64450		*yegD*	Chaperones / Heat shock proteins
PA14_64480	y	*osmE*	Membrane proteins; Adaptation / Protection
PA14_64490	y		Hypothetical, unclassified or unknown
PA14_64500	y		Transcriptional regulators
PA14_64920	y		Adaptation / Protection; Chemotaxis
PA14_65410	y	*orn*	Transcription, RNA processing and degradation
PA14_66570	y	*gltB*	Amino acid biosynthesis and metabolism
PA14_66580	y		Protein secretion / Export apparatus
PA14_66620	y	*pilQ*	Motility / Attachment

PA14_66850	y	*phaD*	Transcriptional regulators
PA14_67130	y	*ybeJ*	Transport of small molecules
PA14_67600	y	*glnA*	Amino acid biosynthesis and metabolism
PA14_68930	y		Transport of small molecules; Membrane proteins
PA14_69050	y		Hypothetical, unclassified or unknown
PA14_69220	y	*ppx*	Nucleotide biosynthesis and metabolism; Adaptation / Protection
PA14_69350	y		Hypothetical, unclassified or unknown
PA14_69510	y		Hypothetical, unclassified or unknown
PA14_69870		*pchP*	Putative enzymes
PA14_69940	y	*xpt*	Nucleotide biosynthesis and metabolism
PA14_70080	y	*lrp*	Central intermediary metabolism; Transcriptional regulators
PA14_70730		*ubiA*	Energy metabolism; Biosynthesis of cofactors, prosthetic groups and carriers
PA14_71340			Hypothetical, unclassified or unknown
PA14_71360			Hypothetical, unclassified or unknown
PA14_71370			Hypothetical, unclassified or unknown
PA14_71640	y		Transcriptional regulators
PA14_71750			Transcriptional regulators
PA14_71900	y		Hypothetical, unclassified or unknown
PA14_72250	y		Translation, post-translational modification and degradation
PA14_72340	y	*gltP*	Membrane proteins; Transport of small molecules
PA14_72350			Hypothetical, unclassified or unknown
PA14_72590		*znuB*	Membrane proteins; Transport of small molecules
PA14_72890	y		Transcriptional regulators
PA14_72990			Hypothetical, unclassified or unknown
PA14_73140	y		Fatty acid and phospholipid metabolism; Putative enzymes
PA14_73390			Hypothetical, unclassified or unknown
PA1838-PA1839	y	*sRNA1059*	Non-coding RNA gene (sRNA)
PA2750-PA2751	y	*sRNA1559*	Non-coding RNA gene (sRNA)
PA3001-PA3002	y	*sRNA1714*	Non-coding RNA gene (sRNA)
PA4272.1	y	*P27*	Non-coding RNA gene (sRNA)

PA4704.2		*prrF2*	Non-coding RNA gene (sRNA)
PA4726.11	y	*crcZ*	Non-coding RNA gene (sRNA)
PA4726.2		*P30*	Non-coding RNA gene (sRNA)
PA5181.1	y	*P34*	Non-coding RNA gene (sRNA)
PA5316.1		*102/16*	Non-coding RNA gene (sRNA)

Table S2. Genes that exhibited a differential in antisense expression under changing environments and upon overexpression and/or inactivation of an alternative sigma factor

**Gene locus**	**Gene name**	**Antisense regulation by sigma factor (Schulz et al. 2015)**	**Antisense primary Regulon (Schulz et al. 2015)**	**Sense primary regulon (Schulz et al. 2015)**	**Differential expression under environmental growth conditions (Dotsch et al. 2015) (most influential are listed)**	**PseudoCAP functional class**
PA14_00010	*dnaA*	FecI2↑	FecI2	RpoD,RpoS	iron, heat50, trans	DNA replication, recombination, modification and repair
PA14_01320	*coIII*	RpoS↓	RpoS	RpoS,SigX	trans, stat, ex-vivo	Energy metabolism
PA14_07380		RpoN↓			trans, stat, heat42/50	Hypothetical, unclassified or unknown
PA14_07730	*ksgA*	AlgU↑, RpoH↑, RpoN↑	RpoN		almost all conditions	Transcription, RNA processing and degradation

PA14_08540		SigX↑	SigX		osmo, ex-vivo, iron	Cell wall / LPS / capsule
PA14_15580		RpoH↑	RpoH		heat42/50, stat, ex-vivo	Putative enzymes
PA14_16670	*cadR*	RpoS↓	RpoS		stat, trans, heat42/50	Transcriptional regulators
PA14_17720		RpoH↑		FliA	almost all conditions	Transcriptional regulators
PA14_22140		RpoH↑		RpoH	heat42/50, exp, trans	Hypothetical, unclassified or unknown
PA14_23200	*yciK*	RpoH↑			heat42/50, iron, exp	Putative enzymes
PA14_27830		RpoH↑			heat42/50, osmo, exp	Transcriptional regulators; Adaptation / Protection
PA14_28180	*ordL*	RpoN↓	RpoS	RpoD	almost all conditions	Energy metabolism; Carbon compound catabolism
PA14_28750		RpoN↑	RpoN	RpoD	trans, stat, anox	Hypothetical, unclassified or unknown
PA14_36470		RpoN↑	RpoN		almost all conditions	Hypothetical, unclassified or unknown
PA14_37730		RpoH↑			heat42/50, stat, exp	Transport of small molecules; Membrane proteins
PA14_40070	*gst*	AlgU↓			heat42/50, anox, stat	Central intermediary metabolism; Putative enzymes
PA14_40080		RpoS↓	RpoS		stat, trans, osmo	Hypothetical, unclassified or unknown
PA14_40220		RpoS↓			almost all conditions	Putative enzymes
PA14_44470	*hemN*	FliA↑	FliA,RpoN		exp, heat42/50, trans	Biosynthesis of cofactors, prosthetic groups and carriers
PA14_44640		RpoS↓			att, stat, trans	Hypothetical, unclassified or unknown
PA14_46620		FecI2↑,<br>RpoH↑	FliA,<br>RpoH	RpoN	heat42, iron, anox	Putative enzymes
PA14_46910	*ybeJ*	RpoH↑	RpoH	RpoN,FecI	anox, heat42, trans	Transport of small molecules

PA14_48700	*kefB*	RpoH↑	RpoH	SigX	almost all conditions	Transport of small molecules
PA14_51340	*mvfR*	FpvI↑			almost all conditions	Biosynthesis of cofactors, prosthetic groups and carriers; Transcriptional regulators
PA14_51350	*phnB*	PvdS↑			almost all conditions	Amino acid biosynthesis and metabolism; Adaptation / Protection
PA14_51540		RpoH↑			heat42/50, anox, att	Related to phage, transposon or plasmid
PA14_51890		RpoN↓	RpoN,SigX		almost all conditions	Hypothetical, unclassified or unknown
PA14_55820		RpoS↓		FliA,RpoN	almost all conditions	Membrane proteins; Motility / Attachment
PA14_56170		RpoN↓		RpoD	heat42/50, stat, trans	Hypothetical, unclassified or unknown
PA14_59370		RpoN↓	RpoN	FliA	heat42/50, stat, trans	Hypothetical, unclassified or unknown
PA14_61040	*katB*	RpoH↑	RpoH	RpoH	heat42/50, stat, osmo	Adaptation / Protection
PA14_61820	*ychF*	PvdS↑	RpoH	RpoN	iron, exp, heat42/50	Translation, post-translational modification and degradation
PA14_62160	*ilvI*	RpoH↑	RpoH	RpoD	heat42/50, osmo, stat	Biosynthesis of cofactors, prosthetic groups and carriers; Amino acid biosynthesis and metabolism
PA14_64400		RpoH↑, RpoN↑	RpoH		stat, heat42/50, trans	Hypothetical, unclassified or unknown
PA14_64450	*yegD*	RpoS↓	RpoS,AlgU		anox, stat, trans	Chaperones / Heat shock proteins
PA14_64490		RpoN↓	AlgU	AlgU	almost all conditions	Hypothetical, unclassified or unknown
PA14_64500		RpoN↓	AlgU		almost all conditions	Transcriptional regulators
PA14_66570	*gltB*	RpoH↑			heat42/50, ex-vivo, trans	Amino acid biosynthesis and metabolism

PA14_66580		SigX↑	SigX	AgU,FliA	osmo, trans, heat42/50	Protein secretion / Export apparatus
PA14_67130	*ybeJ*	RpoH↑	RpoH	FecI	heat42/50, anox, stat	Transport of small molecules
PA14_67600	*glnA*	SigX↑	AlgU	RpoN	osmo, anox, trans	Amino acid biosynthesis and metabolism
PA14_70730	*ubiA*	RpoS↓	RpoS		anox, stat, trans	Energy metabolism; Biosynthesis of cofactors, prosthetic groups and carriers
PA14_72890		AlgU↓	AlgU	RpoD	heat42/50, ex-vivo, trans	Transcriptional regulators
PA3001-PA3002	sRNA1714	RpoN↑	AlgU		almost all conditions	Non-coding RNA gene (sRNA)
PA4726.2	P30	RpoN↓	RpoN	RpoH,RpoN	anox, stat, trans	Non-coding RNA gene (sRNA)

Table S3: Overlap between the genes having asRNA transcripts found in this and 3 previous studies (32-34)

**Gene locus**	**Gene name**	**Wurtzel et al.**	**Gomez-Lozano et al.**	**Ferrara et al.**	**This study**
PA0296-PA0297	*P1*			y	y
PA0836.1	*P5*			y	y
PA14_00010	*dnaA*	y	y		y
PA14_00480			y	y	y
PA14_01320	*colII*			y	y
PA14_01330				y	y
PA14_01600	*mmsA*	y			y

PA14_01970	*triC*		y	y	
PA14_03190		y	y		
PA14_03420		y	y		y
PA14_04080	*yecS*		y		y
PA14_04820		y		y	
PA14_05310	*gshB*	y	y	y	y
PA14_05520	*mexR*	y			y
PA14_06030			y		y
PA14_06870	*dnr*	y			y
PA14_07770	*ostA*		y		y
PA14_08540			y	y	y
PA14_08750	*rplL*			y	y
PA14_08850	*rplC*	y			y
PA14_09050	*secY*	y			y
PA14_09160	*bfrA*			y	y
PA14_09970	*fpvB*	y	y		
PA14_10540	*fixG*		y		y
PA14_10570	*hpcH*	y	y		y
PA14_13940			y		y
PA14_13950		y			y
PA14_15090				y	y
PA14_15140			y		y
PA14_15360		y			y
PA14_15580		y			y
PA14_16380			y		y
PA14_19370	*asnB*		y	y	y
PA14_20510		y	y		
PA14_21210		y		y	y
PA14_21340	*fadD2*	y	y		
PA14_21840		y			y

PA14_22180		y			y
PA14_22270		y		y	y
PA14_22480		y	y		y
PA14_22800	*yciB*	y	y		y
PA14_23100	*mutT*			y	y
PA14_23470	*wbpM*	y			y
PA14_24650	*rmf*	y	y		y
PA14_25840		y	y		y
PA14_27630	*rebB1*	y			y
PA14_27770	*yadG*	y			y
PA14_28290		y		y	
PA14_28400	*oprQ*		y		y
PA14_28410			y	y	y
PA14_28610				y	y
PA14_29600	*ptpS*		y		y
PA14_30020	*nuoA*	y	y	y	
PA14_30110	*purB*	y			y
PA14_30280	*trxB1*	y	y		
PA14_30700		y			y
PA14_30960	*traG*	y			y
PA14_30980		y			y
PA14_30990		y			y
PA14_33360				y	y
PA14_35710				y	y
PA14_35720		y		y	y
PA14_37060	*cupA1*	y	y		
PA14_37200		y			y
PA14_39480		y			y
PA14_39520			y	y	
PA14_40080		y			y

PA14_40740				y	y
PA14_40750				y	y
PA14_41680	*ydiA*	y	y		y
PA14_45010	*hyi*	y			y
PA14_45950	*rsaL*			y	y
PA14_45960	*lasR*		y	y	y
PA14_46150		y		y	y
PA14_46460		y		y	y
PA14_46910	*ybeJ*		y		y
PA14_48530		y	y		y
PA14_50000		y		y	y
PA14_50510		y		y	y
PA14_51240	*purC*	y		y	y
PA14_51340	*mvfR*	y			y
PA14_51500		y			y
PA14_51540		y		y	y
PA14_51880	*oprD*	y			y
PA14_52465		y			y
PA14_52580	*lysC*		y		y
PA14_53570		y			y
PA14_53650			y		y
PA14_53800	*mntH2*	y			y
PA14_54830			y		y
PA14_54850		y			y
PA14_54930		y			y
PA14_55410		y			y
PA14_55820		y			y
PA14_56560		y			y
PA14_57260	*lpxC*			y	y
PA14_58060			y	y	y

PA14_58550	*lpxO1*		y		y
PA14_58910		y			y
PA14_59090		y			y
PA14_59180		y			y
PA14_59350	*pilV2*	y			y
PA14_59370		y		y	y
PA14_59580		y		y	
PA14_59770	*rcsB*	y			y
PA14_59780	*rcsC*	y			y
PA14_59790	*pvrR*	y			y
PA14_59830		y			y
PA14_59840		y		y	y
PA14_60050		y			y
PA14_60310	*pilY1*	y	y		y
PA14_60760		y	y		
PA14_61040	*katB*	y			y
PA14_61820	*ychF*	y			y
PA14_62160	*ilvI*		y		y
PA14_62480	*aspC*	y	y	y	
PA14_64490			y		y
PA14_66570	*gltB*	y			y
PA14_66580		y			y
PA14_66620	*pilQ*	y		y	y
PA14_66670	*ponA*	y	y	y	
PA14_68850	*gcvP1*		y	y	
PA14_69050		y		y	y
PA14_69220	*ppx*	y			y
PA14_69510		y			y
PA14_71900		y	y	y	y
PA14_71960	*wzm*	y	y		

PA14_72350			y	y	y
PA14_72480	*ysxC*	y	y	y	
PA14_72890			y	y	y
PA14_73140		y			y
PA1838-PA1839	*sRNA159*			y	y
PA4726.11	*crcZ*			y	y
PA4726.2	*P3*			y	y
